# Global, regional, and national trends and burden of hypertensive disorders in pregnancy among women of childbearing age from 1990 to 2021

**DOI:** 10.3389/fgwh.2025.1533843

**Published:** 2025-05-09

**Authors:** Zhongyun Tang, Chao Ma, Jin Liu, Chongdong Liu

**Affiliations:** ^1^ Department of Obstetrics and Gynecology, Beijing Chao-Yang Hospital, Capital Medical University, Beijing, China; ^2^ Clinical Medical College, Hebei University, Baoding, Hebei, China

**Keywords:** GBD 2021, women of childbearing age, disease burden, hypertensive disorders in pregnancy, DALYs

## Abstract

**Background:**

Hypertensive disorders of pregnancy (HDP) are a significant cause of maternal and perinatal morbidity and mortality worldwide. This study aims to use the Global Burden of Disease 2021 database to analyze the prevalence trends and disease burden of HDP across the globe from 2019 to 2021.

**Methods:**

We analyzed four key metrics related to HDP (prevalence, incidence, mortality, and DALYs) using data from the GBD Database. Trends were assessed using the estimated annual percentage change (EAPC) and changes in disease burden.

**Results:**

In 2021, global HDP prevalence cases, incidence cases, mortality cases, and DALYs were 3.51 million, 18.00 million, 37.58 million, and 2.44 million, respectively, with percentage changes of 14%, 15%, −29%, and −29% over the study period. Prevalence and incidence rates increased (EAPCs: −0.7 and −0.67), while mortality and DALYs rates decreased (EAPCs: −2.29 and −2.28). Low Socio-demographic Index (SDI) regions had the highest HDP burden, accounting for about half of the global total. The 25–29 age group had the highest incidence cases.

**Conclusion:**

Over the past 32 years, HDP prevalence cases and incidence cases have risen globally, but death cases and DALYs cases have significantly decreased, particularly in low SDI regions and the 25–29 age group. The global HDP burden is higher in regions with lower SDI. Our findings highlight regional and age-related disparities in HDP, providing a basis for targeted interventions and prevention strategies.

## Introduction

Hypertensive disorders of pregnancy (HDP) encompass a spectrum of conditions characterised by the coexistence of pregnancy and hypertension, which can precipitate substantial maternal and fetal mortality (1). These disorders are clinically stratified based on the temporal onset of hypertension and the severity of the clinical manifestations (2). Globally, HDP exhibits a prevalence that varies significantly across different regions. Literature reviews indicate HDP global prevalence is between 5 and 10% (3), a prevalence of 7% in the United States (4), 5%–6.4% in China (5), and notably higher rates of 10% in economically disadvantaged areas of Africa (6).

HDP exert a plethora of detrimental effects on both the mother and the fetus. Maternal consequences are particularly severe, with pre-eclampsia and eclampsia posing significant risks for placental abruption, seizures, cerebrovascular accidents, multi-organ dysfunction, and, in extreme cases, maternal mortality (7, 8). A meta-study showed that patients with HDP were 3.6 times more likely than normal women to develop chronic hypertension (9). Additionally, chronic hypertension is associated with long-term damage to the renal and cardiovascular systems (10). Fetal repercussions are predominantly characterised by intrauterine growth restriction, preterm birth, fetal hypoxia, and the potential for stillbirth (11, 12). Advanced maternal age is a significant predictor of hypertensive disorders during pregnancy, with women over 35 years of age exhibiting a twofold increased risk compared to their younger counterparts (13). Additionally, nutritional factors, particularly diets deficient in calcium and magnesium (14), have been implicated in the mechanism of pregnancy-associated hypertension.

The Global Burden of Disease (GBD) 2021 Database (https://ghdx.healthdata.org/gbd-2021) encompasses a comprehensive array of data spanning from 1990 to 2021; HDP has significantly impacted global health, as evidenced by a substantial rise in global incidence from 16.3 million cases in 1990 to 18.08 million in 2019, reflecting a 10.92% increase. In 2019, there were 27,834 HDP-related deaths, marking a 30.05% decrease from 1990 levels (15). Acknowledging the variability in HDP incidence and mortality rates, appositional status, and limited access to quality healthcare further exacerbate the risk, especially in different countries and regions, within populations experiencing economic disadvantages, underscoring the need for tailored public health strategies. Currently, there is no global consensus on the diagnostic criteria for HDP resulting in inconsistent diagnostic standards across different regions. This inconsistency poses certain challenges when assessing the global incidence, prevalence, mortality, and disability-adjusted life years (DALYs) of HDP. Therefore, when assessing the disease burden in low SDI (Socio-demographic Index) and middle SDI regions, it is precisely because of the lack of uniform diagnostic criteria that leads to an uneven distribution of the disease burden within each region (16). A meta-analysis found that low- and middle-income countries face significant constraints and barriers to the management of HDP, such as insufficient therapeutic medications, lack of diagnostic reagents, insufficient infrastructure, and insufficient knowledge of treatment among healthcare professionals (17). The impact of HDP extends to critical global health indicators, including DALYs, years of life lost due to premature mortality (YLLs), and years lived with disability (YLDs) (15, 16).

The detrimental impact of HDP on maternal and infant health is well recognized. However, research on its contribution to the global burden of disease remains relatively limited. This study aims to address this gap by conducting an in-depth analysis of the most recent data from the GBD 2021 database. It provides a detailed examination of the disease burden of HDP across different regions and age groups worldwide, thereby offering more targeted and scientifically sound evidence for research in this field and the formulation of public health policies.

## Methods

### Data sources and disease definition

The GBD 2021 database provides annual estimates of incidence and mortality for 371 diseases and injuries across 204 countries and territories from 1990 to 2021 (18). The SDI is a standardized composite measure ranging from 0 to 1 that allows comparisons across geographic regions and time. It is calculated based on national per capita income, average years of education among individuals aged 15 and older, and total fertility rate, and categorizes countries and regions into five levels: low, low-middle, middle, high-middle, and high. The GBD 2021 database is widely used to assess the global burden of health issues, analyze trends in diseases and risk factors, and provide data support for public health policy-making. HDP refer to hypertension that arises after 20 weeks of gestation and returns to normal within 12 weeks postpartum. The diagnostic criterion is typically a blood pressure of ≥140/90 mmHg, corresponding to ICD-10 code O13.

### DALYs

DALYs are a comprehensive measure of disease burden, combining YLLs and YLDs. One DALY represents one lost year of healthy life. YLLs are calculated based on the number of deaths and standard life expectancy loss, while YLDs are derived from the number of incident cases, disability weights, and the duration of the condition. DALYs are widely used to assess the impact of diseases and injuries on population health and are a core metric in the GBD study. They provide a basis for health policy decisions, resource allocation, and evaluation of the cost-effectiveness of public health interventions (16).

### Estimated annual percent change

Estimated Annual Percent Change (EAPC) is a statistical metric used to measure the trend in disease indicators over a specific period. It is calculated through linear regression analysis and reflects the rate of increase or decrease in a given metric. Unlike simple comparisons between the starting and ending years, EAPC offers a more detailed understanding of time trends, helping researchers and public health professionals more accurately assess changes in disease burden (16).

## Results

### Global level

**Figure 1:**
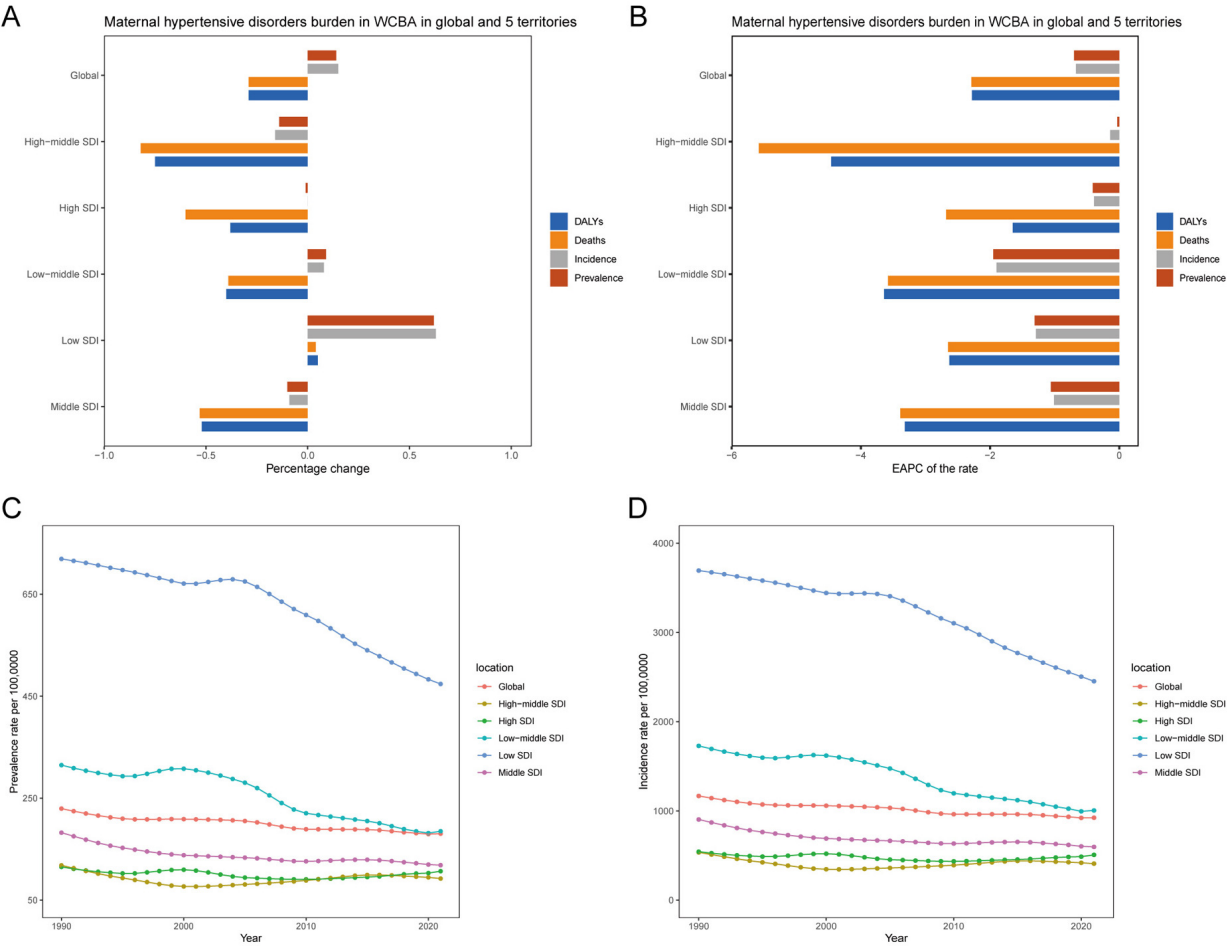
Global and regional temporal trends in HDP burden within WCBA. **(A)** Percentage changes in the number of prevalent cases, incident cases, deaths, and DALYs from 1990 to 2021. **(B)** EAPC in prevalence, incidence, mortality, and DALY rates from 1990 to 2021. **(C)** Prevalence rates per 100,000 individuals from 1990 to 2021 in global and five distinct territories. **(D)** Incidence rates per 100,000 individuals from 1990 to 2021 in global and five distinct territories.

Globally, there has been a notable escalation in the prevalence cases, incidence cases, mortality cases, and DALYs cases in HDP. Specifically, the prevalence of HDP cases has risen from 3.07 million in 1990 to 3.51 million in 2021, marking a 14% increase. Similarly, the incidence of HDP cases has shown a 15% increase, escalating from 15.61 million in 1990 to 18.00 million in 2021. Conversely, mortality cases have significantly declined, decreasing from 53.17 thousand in 1990 to 37.58 thousand in 2021, corresponding to a 29% reduction. Moreover, the burden of HDP, as measured by DALYs, has also witnessed a substantial decrease, plummeting from 3.44 million in 1990 to 2.44 million in 2021, indicating a 29% reduction (Table 1 and Supplementary Tables S1–S3, Figure 1A). Contrary to the global trend, the prevalence rates, incidence rates, mortality rates and DALYs rates of HDP within WCBA declined from 1990 to 2021. EAPC for these metrics was −0.7 (−0.76 to −0.64), −0.67 (−0.73 to −0.62), −2.29 (−2.33 to −2.24), and −2.28 (−2.33 to −2.23). These figures underscore a significant reduction in the epidemiological burden of HDP in the WCBA, contradicting the global escalation observed elsewhere (Table 1 and Supplementary Tables S1–S3, Figure 1A).

**Table 1:** The prevalence of hypertensive disorders of pregnancy cases and rates among WCBA in 1990 and 2021, and the trends from 1990 to 2021.

Location	1,990_thousands(95% UI)	2021_thousands(95% UI)	Percentage change	1,990_per 100 000 (95% UI)	2021_per 100 000 (95% UI)	EAPC(95% CI)
Global	3,068.92 (1,991.74–4,471.58)	3,513.83 (2,274.9–5,074.4)	0.14	229.48 (148.93–334.36)	180.3 (116.73–260.38)	−0.7 (−0.76–0.64)
High SDI	261.12 (166.58–391.15)	259.43 (167.82–377.42)	−0.01	115.18 (73.48–172.54)	106.69 (69.02–155.22)	−0.41 (−0.63–0.19)
High-middle SDI	328.12 (206.58–499.32)	281.66 (176.68–418.28)	−0.14	118.13 (74.38–179.77)	92.31 (57.9–137.08)	−0.03 (−0.47–0.4)
Middle SDI	815.09 (524.86–1,198.1)	733.2 (466.1–1,069.54)	−0.1	182.32 (117.4–267.99)	118.55 (75.36–172.93)	−1.06 (−1.23–0.89)
Low-middle SDI	859.18 (553.33–1,264.3)	937.05 (608.83–1,358.54)	0.09	314.81 (202.75–463.26)	185.09 (120.26–268.34)	−1.95 (−2.19–1.71)
Low SDI	803.3 (527.56–1,169.11)	1,300.2 (859.18–1,900.01)	0.62	719.25 (472.37–1,046.79)	473.99 (313.21–692.65)	−1.31 (−1.47–1.14)
Andean Latin America	10.25 (6.85–14.76)	18.06 (12.57–24.81)	0.76	108.04 (72.28–155.6)	103.5 (72.05–142.13)	−0.21 (−0.54–0.13)
Australasia	8.03 (5.35–11.68)	7.4 (4.51–10.92)	−0.08	149.66 (99.6–217.63)	102.55 (62.51–151.26)	−1.13 (−1.43–0.82)
Caribbean	18.48 (11.89–29.23)	15.57 (9.79–23.36)	−0.16	198.28 (127.52–313.57)	129.41 (81.41–194.22)	−1.4 (−1.52–1.28)
Central Asia	14.37 (8.95–22.07)	19.09 (11.95–28.72)	0.33	85.67 (53.33–131.57)	78.68 (49.25–118.34)	0.41 (0.01–0.82)
Central Europe	25.1 (15.37–39.87)	17.85 (11.48–25.98)	−0.29	81.72 (50.06–129.82)	69.31 (44.59–100.88)	−0.21 (−0.51–0.08)
Central Latin America	114.31 (73.92–167.95)	101.87 (68.49–145.2)	−0.11	272.72 (176.36–400.7)	149.38 (100.43–212.92)	−1.01 (−1.44–0.57)
Central Sub-Saharan Africa	113.94 (75.08–166.82)	176.2 (114.29–257.23)	0.55	921.72 (607.39–1,349.53)	539.63 (350.02–787.76)	−1.58 (−1.74–1.43)
East Asia	276.17 (166.21–440.21)	155.82 (94.66–238.2)	−0.44	82.84 (49.86–132.04)	47.09 (28.61–71.98)	−0.9 (−2.35–0.57)
Eastern Europe	102.02 (62.97–156.52)	88.15 (54.96–134.27)	−0.14	184.51 (113.88–283.07)	182.73 (113.93–278.32)	1.43 (0.84–2.02)
Eastern Sub-Saharan Africa	387.72 (254.12–568.69)	600.94 (395.64–874.53)	0.55	898.53 (588.91–1,317.93)	561.1 (369.41–816.54)	−1.4 (−1.57–1.23)
High-income Asia Pacific	31.57 (19.28–48.83)	18.74 (12.08–27.29)	−0.41	69.03 (42.15–106.76)	49.26 (31.76–71.73)	−1.86 (−2.43–1.29)
High-income North America	123.65 (77.64–192.38)	130.94 (85.85–185.02)	0.06	166.27 (104.4–258.69)	155.84 (102.17–220.21)	−0.33 (−0.54–0.12)
North Africa and Middle East	238.75 (150.94–351.38)	269.59 (165.66–404.6)	0.13	305.6 (193.2–449.76)	169.19 (103.96–253.92)	−1.31 (−1.5–1.13)
Oceania	3.72 (2.35–5.53)	6.84 (4.28–10.39)	0.84	239.24 (151.41–355.64)	197.15 (123.21–299.47)	−0.78 (−0.83–0.73)
South Asia	688.77 (430.65–1,055.82)	596.66 (373.65–900.94)	−0.13	270.22 (168.95–414.22)	120.75 (75.62–182.33)	−3.14 (−3.69–2.59)
Southeast Asia	249.71 (155.64–369.78)	244.65 (152.74–363.92)	−0.02	207.76 (129.5–307.67)	133.54 (83.37–198.64)	−1.39 (−1.46–1.32)
Southern Latin America	24.66 (15.38–37.58)	30.92 (20.39–44.9)	0.25	199 (124.08–303.22)	177.37 (116.99–257.59)	−0.11 (−0.23–0.01)
Southern Sub-Saharan Africa	72.05 (47.2–104.36)	74.28 (48.45–106.56)	0.03	542.08 (355.14–785.17)	342.1 (223.15–490.77)	−1.21 (−1.3–1.12)
Tropical Latin America	74.58 (46.34–118.45)	65.03 (42.65–93.8)	−0.13	186.97 (116.18–296.94)	107.29 (70.36–154.76)	−1.76 (−2.17–1.36)
Western Europe	82.62 (51.16–127.5)	85.58 (53.3–127.78)	0.04	86.47 (53.55–133.44)	91.85 (57.2–137.13)	0.21 (0.01–0.42)
Western Sub-Saharan Africa	408.44 (272.31–586.92)	789.65 (527.56–1,130.13)	0.93	936.69 (624.49–1,345.99)	658.64 (440.03–942.64)	−0.91 (−1.16–0.66)

It indicated that the growth of the burden of disease is slowing down and may even be on the decline in certain regions or populations. This may be closely related to the implementation of public health measures, advances in medical technology or improvements in socio-economic conditions.

### SDI regional level

In 2021, low SDI regions had the highest absolute HDP across prevalence cases, incidence cases, mortality cases and DALYs cases in WCBA. Specifically, these regions accounted for 1.30 million prevalent cases (0.86–1.90), 6.73 million incident cases (5.70–7.94), 18.31 thousand mortality cases (14.98–22.31), and 1.18 million DALYs cases (0.97–1.43). Notably, these figures constituted approximately 50% of the global HDP burden (Table 1 and Supplementary Tables S1–S3, Figures 1C,D and Supplementary Figures S1A,B). Prevalence cases, incidence cases, mortality cases, and DALYs cases of HDP showed a progressive decline with decreasing SDI. The low SDI regions experienced the most significant percentage changes in prevalence cases and incidence cases, approximately 60%. In contrast, the high-middle SDI regions demonstrated the largest percentage changes in mortality cases and DALYs cases, around 75%. Conversely, the high SDI regions had the smallest percentage changes in prevalence cases and incidence cases, approximately 1%, and the low SDI regions showed the least change in mortality cases and DALYs cases, approximately 5% (Table 1 and Supplementary Table S1–S3, Figure 1A). Significantly, between 1990 and 2021, the low-middle SDI region exhibited a marked decline in both the prevalence rates and incidence rates of HDP, with an EAPC of −1.95 (−2.19 to −1.71) and −1.9 (−2.08 to −1.73). In parallel, the high-middle SDI region witnessed a pronounced reduction in mortality rates and DALY rates, with an EAPC of −5.58 (−5.68 to −5.48) and −4.46 (−4.57 to −4.36) (Table 1 and Supplementary Tables S1–S3, Figure 1B). In 2021, regions with low and low-middle SDI demonstrated relatively elevated prevalence rates, incidence rates, mortality rates, and DALYs rates associated with HDP. Concurrently, the low SDI region experienced the most rapid decline in prevalence rates and incidence rates, while the high-middle SDI region showed the steepest decrease in mortality rates and DALY rates.

The results indicate that low SDI regions bear the heaviest burden in terms of prevalence cases, incidence cases, mortality cases, and DALYs cases, while high SDI regions have the lightest burden. This suggests that the global burden of HDP is closely related to the SDI.

### GBD regional level

**Figure 2:**
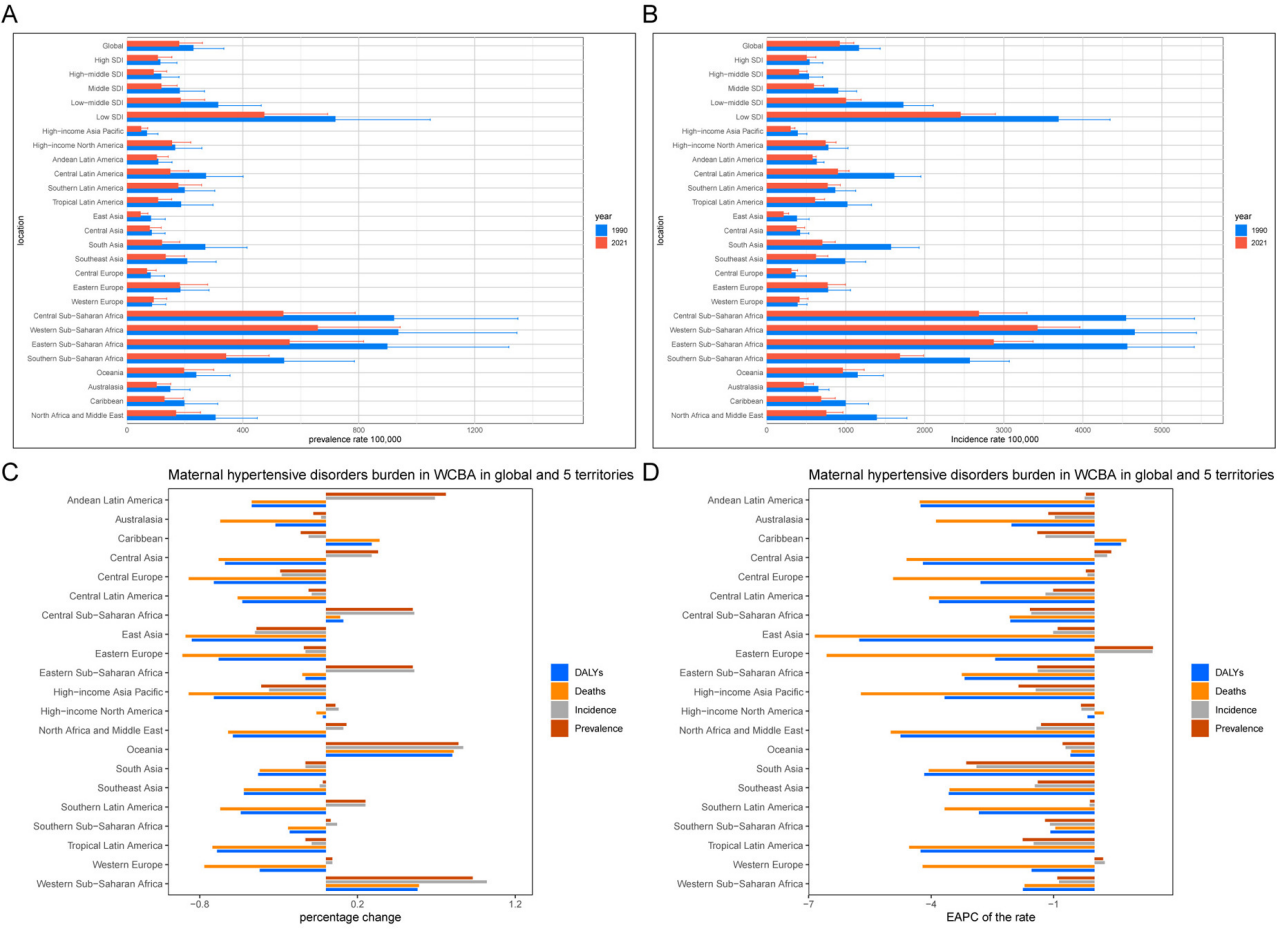
Regional temporal trends in HDP burden within WCBA. **(A)** Prevalence rates per 100,000 population in 1990 and 2021. **(B)** Incidence rates per 100,000 population in 1990 and 2021. **(C)** Percentage changes in the number of prevalent cases, incident cases, deaths, and DALYs from 1990 to 2021. **(D)** EAPC in prevalence, incidence, mortality, and DALY rates from 1990 to 2021.

Over the period from 1990 to 2021, WCBA experienced a temporal increase in the absolute numbers of prevalence cases, incidence cases, mortality cases and DALYs cases associated with HDP, with these increases observed in approximately half of the regions. Notably, Andean Latin America, Sub-Saharan Africa, North Africa and Middle East, Central Asia, North Africa and Middle East, Sub-Saharan Africa, Central Asia and Oceania, and Western Europe have demonstrated a consistent upward trend in prevalence cases and incidence cases over the past three decades. Regions such as the Caribbean, Oceania Central and Western Sub-Saharan Africa have shown increasing trends in mortality cases and DALYs cases, all of which are predominantly characterised by low and low-middle SDI regions. Over the past 32 years, the most significant increases in both prevalence rates and DALY rates were observed in Oceania and Western Sub-Saharan Africa, with EAPC for prevalence rates of −0.78 (−0.83 to −0.73) and −0.91 (−1.16 to −0.66), and for DALY rates of −0.59 (−0.78 to −0.39) and −1.75 (−1.84 to −1.66) (Table 1 and Supplementary Tables S1–S3, Figures 2A–D and Supplementary Figures S2A,B). The incidence rates and mortality rates of HDP in WCBA followed a similar pattern to the prevalence rates. The high-income Asia Pacific region has shown a significant downward trend in the absolute cases and rates of prevalence, incidence, mortality, and DALYs of HDP, suggesting a decreasing burden of HDP within this region. This trend is likely closely associated with improvements in healthcare infrastructure and economic conditions.

Overall, these trends reveal significant geographical disparities and dynamic changes in the global burden of HDP. In low and low-middle SDI regions, such as Andean Latin America, Sub-Saharan Africa, and Central Asia, the burden of HDP continues to increase. This may be related to the limited medical resources, weak public health systems, and relatively underdeveloped socio-economic conditions in these areas. In contrast, the HDP burden has significantly decreased in the high-income Asia Pacific region, reflecting progress in healthcare, socio-economic status, and education in that area.

### Countries level

**Figure 3:**
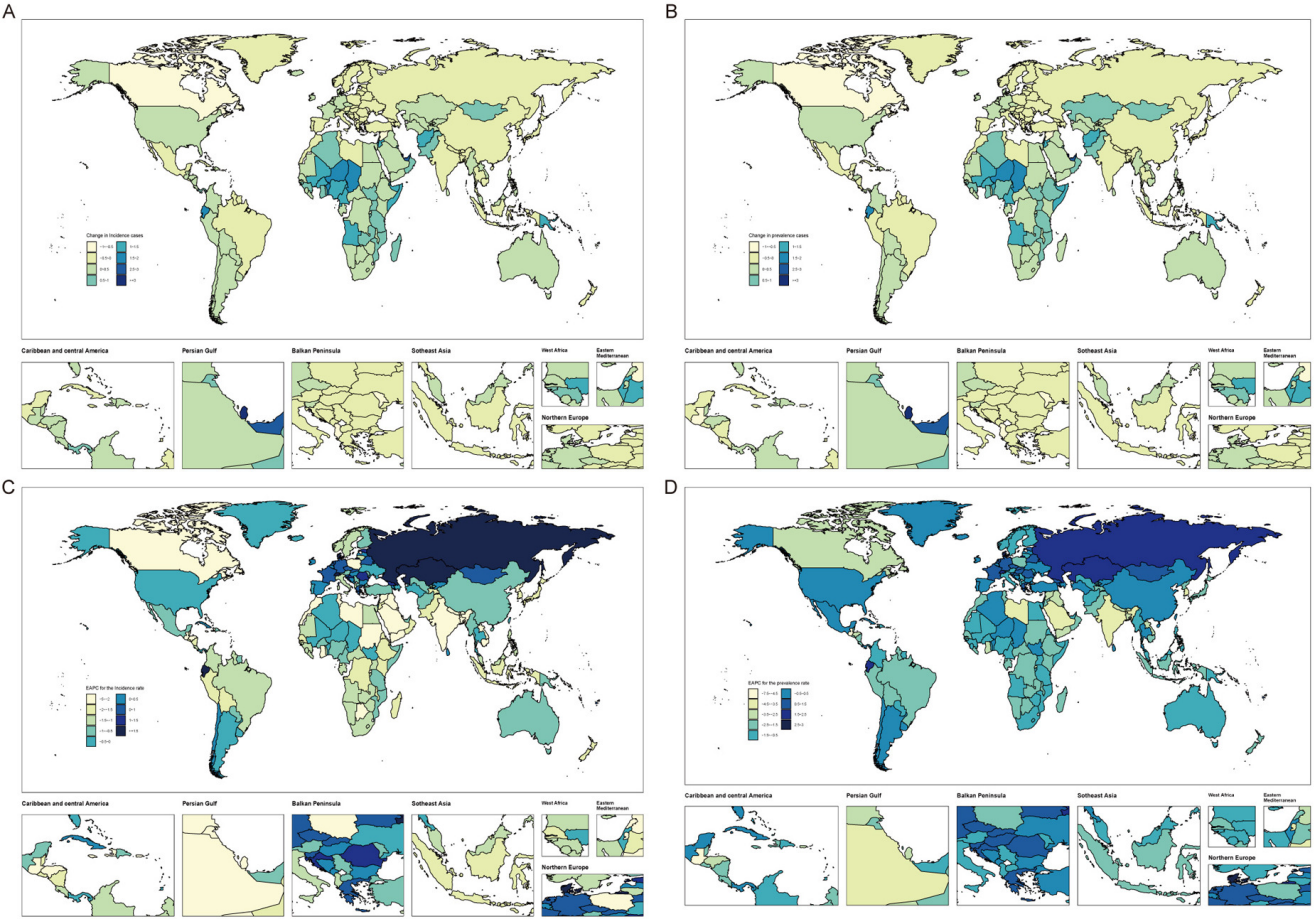
Global temporal trends in HDP burden within WCBA. **(A)** Percentage changes in incident cases across 204 countries from 1990 to 2021. **(B)** Percentage changes in prevalent cases across 204 countries from 1990 to 2021. **(C)** EAPC in incident rates across 204 countries from 1990 to 2021. **(D)** EAPC in prevalent rates across 204 countries from 1990 to 2021.

Between 1990 and 2021, approximately 56% of countries within the WCBA exhibited increasing prevalence cases and incidence cases of HDP. In contrast, only 15% to 20% of these countries displayed an increasing trend in mortality cases and DALYs cases associated with HDP. The most significant increases in prevalence cases and incidence cases were noted in Qatar and the United Arab Emirates, with percentage changes ranging from 273% to 352%. Conversely, the most substantial increases in mortality cases and DALYs cases were observed in Chad and Papua New Guinea, with percentage changes ranging from 101% to 166%. However, the most pronounced decrease in prevalence rates was recorded in Guatemala, a low SDI country, with an EAPC of −7.15 (−7.95 to −6.34). Nepal, also a low SDI country, experienced the most significant decline in incidence rates, with an EAPC of −3.83 (−4.12 to −3.54). The highest decrease in mortality rates was observed in Qatar, a high SDI country, with an EAPC of −9.87 (−10.22 to −9.51). Turkey, a high-middle SDI country, recorded the steepest reduction in DALY rates, with an EAPC of −8.51 (−9.12 to −7.90) (Supplementary Tables S4–S7, Figures 3A–D and Supplementary Figures S3A–D).

The observed data reveal a distinct dichotomy in the epidemiological trends of HDP. Specifically, reductions in prevalence and incidence rates are noted in low SDI regions, whereas declines in mortality rates and DALYs rates are more pronounced in high SDI areas. This divergence underscores the intricate and multifaceted interplay between socio-economic determinants and health outcomes within the context of HDP.

### Age distribution pattern

**Figure 4:**
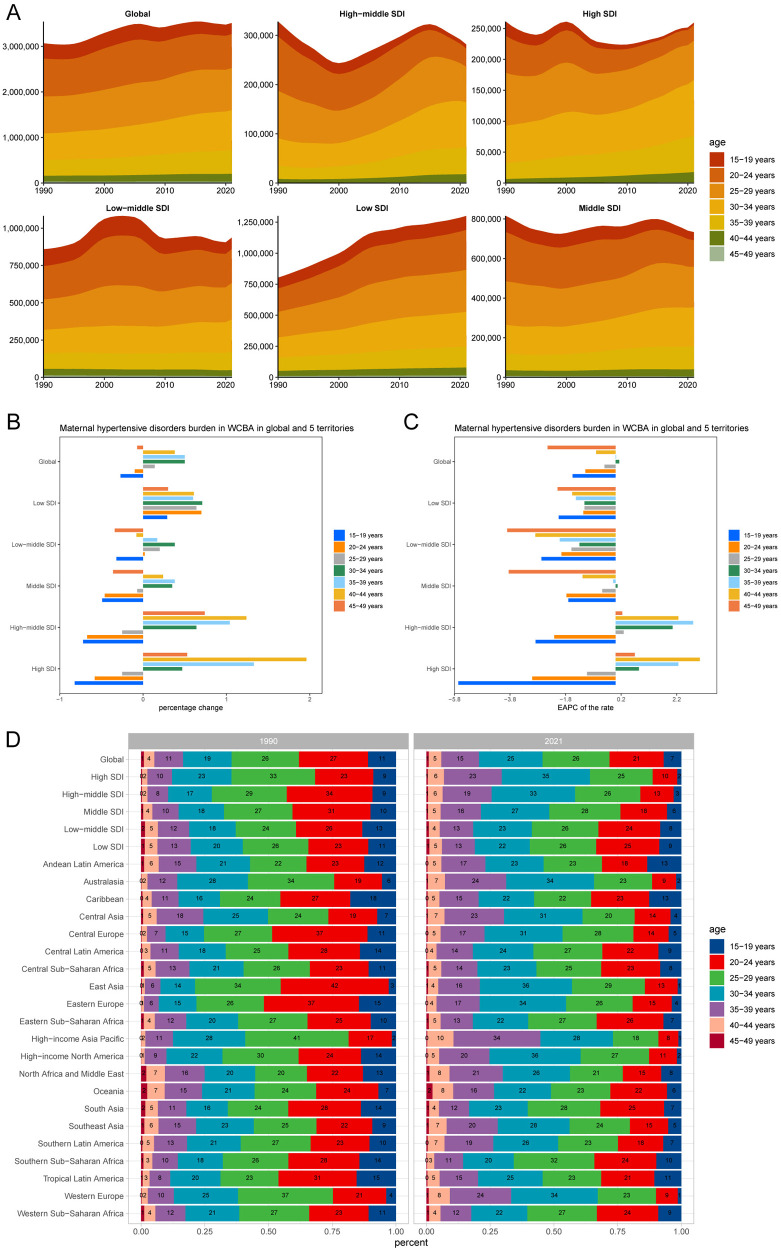
Age-patterned temporal trends in HDP burden within WCBA across different regions. **(A)** Prevalent cases across seven age groups (15–49 years, in 5-year intervals) from 1990 to 2021, globally and in five territories with varying levels of SDI. **(B)** Percentage changes in prevalent cases across seven age groups, globally and in five territories, from 1990 to 2021. **(C)** EAPC in prevalent cases across seven age groups, globally and in five territories, from 1990 to 2021. **(D)** Distribution of prevalent cases across seven age groups as percentages, globally, in five territories, and 21 GBD regions, in 1990 and 2021.

Analysis of the prevalence cases, incidence cases, mortality cases, and DALYs cases number of the percentage change among the WCBA global population reveals significant variations with age (Figure 4A and Supplementary Figures S4A–C). The 45–49 age group demonstrated the lowest percentage changes at 7%, 4%, 0%, and 0%, respectively, for prevalence cases, incidence cases, mortality cases, and DALYs cases. However, the 30–34 age group exhibited the most pronounced percentage changes, with 50%, 50%, 27%, and 20% for prevalence cases, incidence cases, mortality cases, and DALYs cases. These increments are approximately 7–12 times higher than those observed in the 45–49 age group, underscoring the disparate impact of HDP across different age cohorts (Table 2 and Supplementary Tables S8–S10).

**Table 2:** The prevalence of hypertensive disorders of pregnancy cases and rates among WCBA in 1,990 and 2021, and the trends in age patterns.

Location	Age	1,990_thousands(95% UI)	2,021_thousands(95% UI)	Percentage change	1,990_per 100 000 (95% UI)	2,021_per 100 000 (95% UI)	EAPC(95% CI)
Global	15–19 years	337.12 (169.17–586)	245.23 (125.41–422.87)	−0.27 (−0.26–0.28)	131.93 (66.2–229.32)	80.76 (41.3–139.26)	−1.55 (−1.67–1.42)
Global	15–49 years	3,068.92 (1,991.74–4,471.58)	3,513.83 (2,274.9–5,074.4)	0.14 (0.14–0.13)	229.48 (148.93–334.36)	180.3 (116.73–260.38)	−0.7 (−0.76–0.64)
Global	20–24 years	831.76 (432.72–1,428.32)	745.05 (404.16–1,235.75)	−0.1 (−0.07–0.13)	340.69 (177.25–585.05)	253.63 (137.59–420.68)	−1.09 (−1.35–0.83)
Global	25–29 years	811.28 (455.74–1,294.15)	924.01 (548.92–1,404.1)	0.14 (0.2–0.08)	368.6 (207.06–587.98)	317.54 (188.64–482.53)	−0.4 (−0.52–0.27)
Global	30–34 years	584.03 (331.49–903.3)	875.78 (517.53–1,315.25)	0.5 (0.56–0.46)	307.21 (174.37–475.15)	292.97 (173.13–439.98)	0.13 (−0.01–0.28)
Global	35–39 years	346.05 (197.27–544.1)	519.71 (307.19–792.23)	0.5 (0.56–0.46)	199.51 (113.73–313.69)	187.08 (110.58–285.18)	0.02 (−0.17–0.2)
Global	40–44 years	125.76 (71.34–193.4)	173.46 (103.2–260.68)	0.38 (0.45–0.35)	89.68 (50.87–137.92)	69.92 (41.6–105.07)	−0.7 (−0.87–0.53)
Global	45–49 years	32.92 (18.99–48.72)	30.6 (18.18–45.01)	−0.07 (−0.04–0.08)	28.93 (16.69–42.81)	12.99 (7.71–19.1)	−2.45 (−2.62–2.27)
Low SDI	15–19 years	87.53 (43.53–154.02)	112.85 (56.54–198.4)	0.29 (0.3–0.29)	348.32 (173.24–612.91)	183.03 (91.69–321.77)	−2.05 (−2.23–1.88)
Low SDI	15–49 years	803.3 (527.56–1,169.11)	1,300.2 (859.18–1,900.01)	0.62 (0.63–0.63)	719.25 (472.37–1,046.79)	473.99 (313.21–692.65)	−1.31 (−1.47–1.14)
Low SDI	20–24 years	188.77 (99.33–313.23)	321.51 (171.95–530.6)	0.7 (0.73–0.69)	869.09 (457.29–1,442.06)	609.69 (326.09–1,006.22)	−1.16 (−1.36–0.96)
Low SDI	25–29 years	206.52 (120.92–309.5)	338.6 (199.78–507.58)	0.64 (0.65–0.64)	1,114.93 (652.78–1,670.86)	768.68 (453.53–1,152.31)	−1.12 (−1.31–0.93)
Low SDI	30–34 years	164.14 (100.17–240.25)	280.63 (170.98–411.13)	0.71 (0.71–0.71)	1,079.07 (658.52–1,579.43)	756.03 (460.63–1,107.61)	−1.12 (−1.22–1.02)
Low SDI	35–39 years	104.99 (62.51–155.53)	167.6 (100.29–248.01)	0.6 (0.6–0.59)	814.86 (485.17–1,207.08)	525.97 (314.74–778.3)	−1.43 (−1.53–1.34)
Low SDI	40–44 years	39.99 (23.52–58.68)	64.31 (37.82–95.11)	0.61 (0.61–0.62)	403.34 (237.19–591.85)	246.22 (144.78–364.13)	−1.56 (−1.67–1.46)
Low SDI	45–49 years	11.35 (6.68–16.72)	14.7 (8.45–21.86)	0.3 (0.26–0.31)	136.76 (80.47–201.38)	70.8 (40.7–105.25)	−2.09 (−2.18–2)
Low-middle SDI	15–19 years	113.95 (56.5–201.5)	78.05 (39.49–135.38)	−0.32 (−0.3–0.33)	193.95 (96.17–342.96)	86.34 (43.68–149.75)	−2.67 (−2.99–2.34)
Low-middle SDI	15–49 years	859.18 (553.33–1,264.3)	937.05 (608.83–1,358.54)	0.09 (0.1–0.07)	314.81 (202.75–463.26)	185.09 (120.26–268.34)	−1.95 (−2.19–1.71)
Low-middle SDI	20–24 years	222.98 (115.4–385.63)	227.11 (123.5–377.06)	0.02 (0.07–0.02)	427.53 (221.27–739.4)	261.02 (141.94–433.36)	−1.95 (−2.45–1.45)
Low-middle SDI	25–29 years	203.52 (115.22–323.75)	244.59 (144.09–376.57)	0.2 (0.25–0.16)	453.45 (256.71–721.32)	301.05 (177.35–463.49)	−1.59 (−1.88–1.31)
Low-middle SDI	30–34 years	156.98 (86.94–245.47)	215.88 (125.73–330.52)	0.38 (0.45–0.35)	419.23 (232.19–655.58)	292.09 (170.11–447.2)	−1.3 (−1.38–1.22)
Low-middle SDI	35–39 years	105.04 (59.48–169.5)	122.65 (71.14–189.18)	0.17 (0.2–0.12)	327.91 (185.68–529.14)	184.19 (106.83–284.11)	−2.01 (−2.13–1.89)
Low-middle SDI	40–44 years	43.69 (24.29–68.75)	40.19 (23.81–61.44)	−0.08 (−0.02–0.11)	168.41 (93.61–264.98)	69.68 (41.28–106.52)	−2.89 (−2.96–2.81)
Low-middle SDI	45–49 years	13.02 (7.3–19.77)	8.59 (5.12–12.55)	−0.34 (−0.3–0.37)	59.97 (33.62–91.1)	17.37 (10.36–25.39)	−3.9 (−3.97–3.83)
Middle SDI	15–19 years	81.77 (41.33–139.76)	41.36 (22.39–68.75)	−0.49 (−0.46–0.51)	88.68 (44.83–151.57)	47.09 (25.49–78.27)	−1.7 (−1.84–1.56)
Middle SDI	15–49 years	815.09 (524.86–1,198.1)	733.2 (466.1–1,069.54)	−0.1 (−0.11–0.11)	182.32 (117.4–267.99)	118.55 (75.36–172.93)	−1.06 (−1.23–0.89)
Middle SDI	20–24 years	249.23 (129.87–431.7)	134.01 (73.14–222.04)	−0.46 (−0.44–0.49)	282.15 (147.03–488.72)	154.63 (84.39–256.2)	−1.77 (−1.96–1.59)
Middle SDI	25–29 years	218.87 (120.18–352.79)	204.57 (117.75–320.59)	−0.07 (−0.02–0.09)	292.18 (160.44–470.97)	225.8 (129.97–353.85)	−0.48 (−0.66–0.3)
Middle SDI	30–34 years	145.36 (78.87–231.98)	196.56 (112.41–308.27)	0.35 (0.43–0.33)	242.12 (131.37–386.38)	199.04 (113.83–312.16)	0.07 (−0.31–0.45)
Middle SDI	35–39 years	83.94 (46.9–132.75)	116.12 (68.31–182.55)	0.38 (0.46–0.38)	151.43 (84.6–239.48)	126.7 (74.54–199.19)	−0.09 (−0.43–0.26)
Middle SDI	40–44 years	29.21 (16.53–45.36)	36.25 (21.5–55.2)	0.24 (0.3–0.22)	69.13 (39.13–107.35)	44.29 (26.27–67.44)	−1.19 (−1.47–0.9)
Middle SDI	45–49 years	6.72 (3.84–9.99)	4.32 (2.66–6.23)	−0.36 (−0.31–0.38)	19.82 (11.33–29.47)	5.32 (3.28–7.68)	−3.85 (−4.08–3.61)
High-middle SDI	15–19 years	30.33 (14.72–52.61)	8.57 (4.64–14.15)	−0.72 (−0.68–0.73)	64.04 (31.08–111.08)	24.9 (13.48–41.1)	−2.88 (−3.04–2.72)
High-middle SDI	15–49 years	328.12 (206.58–499.32)	281.66 (176.68–418.28)	−0.14 (−0.14–0.16)	118.13 (74.38–179.77)	92.31 (57.9–137.08)	−0.03 (−0.47–0.4)
High-middle SDI	20–24 years	110.48 (56.26–196.48)	36.87 (20.15–61.08)	−0.67 (−0.64–0.69)	229.49 (116.86–408.14)	103.57 (56.61–171.61)	−2.21 (−2.33–2.08)
High-middle SDI	25–29 years	96.34 (51.78–160.11)	71.98 (41.08–114.18)	−0.25 (−0.21–0.29)	210.6 (113.18–350)	178.57 (101.92–283.26)	0.29 (−0.15–0.74)
High-middle SDI	30–34 years	56.22 (28.89–94.02)	92.29 (52.19–145.79)	0.64 (0.81–0.55)	134.66 (69.21–225.21)	179.24 (101.36–283.15)	2.05 (1.51–2.59)
High-middle SDI	35–39 years	26.58 (14.48–45.38)	54.14 (31.86–85.36)	1.04 (1.2–0.88)	67.29 (36.67–114.91)	109.25 (64.29–172.27)	2.79 (2.29–3.3)
High-middle SDI	40–44 years	7.21 (4–11.65)	16.13 (9.82–24.13)	1.24 (1.46–1.07)	23.46 (13–37.89)	35.44 (21.58–53.02)	2.26 (1.7–2.81)
High-middle SDI	45–49 years	0.96 (0.54–1.45)	1.67 (1.06–2.42)	0.74 (0.96–0.67)	3.91 (2.21–5.92)	3.47 (2.2–5.01)	0.24 (−0.15–0.62)
High SDI	15–19 years	23.29 (11.61–38.97)	4.21 (2.34–6.8)	−0.82 (−0.8–0.83)	73.08 (36.42–122.29)	14.49 (8.05–23.38)	−5.66 (−5.82–5.5)
High SDI	15–49 years	261.12 (166.58–391.15)	259.43 (167.82–377.42)	−0.01 (0.01–0.04)	115.18 (73.48–172.54)	106.69 (69.02–155.22)	−0.41 (−0.63–0.19)
High SDI	20–24 years	59.73 (30.62–105.81)	25.09 (14.22–41.83)	−0.58 (−0.54–0.6)	177.87 (91.18–315.11)	79.6 (45.1–132.71)	−3 (−3.31–2.69)
High SDI	25–29 years	85.5 (45.68–140.24)	63.71 (38.25–98.14)	−0.25 (−0.16–0.3)	238.53 (127.43–391.23)	184.35 (110.67–283.96)	−1.03 (−1.24–0.83)
High SDI	30–34 years	60.94 (32.27–100.96)	89.86 (52.28–141.93)	0.47 (0.62–0.41)	171.74 (90.95–284.52)	240.04 (139.67–379.15)	0.84 (0.66–1.01)
High SDI	35–39 years	25.25 (13.63–42.55)	58.82 (34–92.53)	1.33 (1.49–1.17)	75.51 (40.76–127.26)	155.07 (89.64–243.92)	2.26 (2.18–2.35)
High SDI	40–44 years	5.56 (3.16–8.83)	16.43 (10.12–24.66)	1.96 (2.2–1.79)	17.8 (10.11–28.28)	44.75 (27.56–67.18)	3.03 (2.89–3.18)
High SDI	45–49 years	0.85 (0.51–1.24)	1.3 (0.83–1.89)	0.53 (0.63–0.52)	3.36 (2.03–4.92)	3.62 (2.3–5.25)	0.69 (0.12–1.26)

The percentage change in prevalence cases of HDP across the five SDI regions demonstrates distinct age-related trends. In low SDI regions, the prevalence cases initially increase and then decrease with age. In contrast, other SDI regions exhibit a pattern of initial decline, followed by an increase, and eventually a leveling off. For example, in the high SDI region, the percentage change in prevalence cases rises from −0.82 in the 15–19 age group to 1.96 in the 40–44 age group, before declining to 0.53 in the 45–49 age group. The age group with the highest percentage change in prevalence cases peaks at 30–34 years in low and low-middle SDI regions, at 35–39 years in middle SDI regions, and at 40–44 years in high SDI regions. As SDI increases, the percentage change in prevalence of HDP tends to rise with age (Table 2 and Supplementary Tables S8–S10, Figure 4B).

Over the past 32 years, trend analysis from 1990 to 2021 indicates that incidence rates among the 25–49 age groups (25–29 age group, 30–34 age group, 35–39 age group, 40–44 age group, and 45–49 age group) show increasing trends with EAPC of −0.36 (−0.46 to −0.25), 0.1 (−0.04 to 0.24), −0.01 (−0.19 to 0.17), −0.63 (−0.81 to −0.45), and −2.1 (−2.29 to −1.91). Conversely, incidence rates in the 15–24 age groups (15–19 age group and 20–24 age group) exhibit decreasing trends. The 30–39 age group (30–34 age group and 35–39 age group) experienced the highest increase in incidence rates (Supplementary Table S8, Figure 4B and Supplementary Figures S5A,B). In 2021, the 25–29 age group reported the highest incidence cases of HDP globally, with 4.67 million cases (3.58–5.96) and incidence rates of 1605.08 per 100,000 (1,232–2,048.78), surpassing all other age groups (Supplementary Table S8). Furthermore, the 20–24 age group demonstrated the fastest decreases in mortality rates and DALY rates of HDP, with EAPC of −2.47 (−2.63 to −2.31) and −2.39 (−2.56 to −2.23). In contrast, the 40–44 age group showed the fastest increases in mortality and DALY rates, with EAPC of −1.59 (−1.73 to −1.44) and −2.07 (−2.32 to −1.81) (Table 2 and Supplementary Tables S8–S10, Figure 4C and Supplementary Figures S5C–F). This suggests that the burden of HDP was most pronounced in the 40–44 age group.

Among the 21 regions, East Asia exhibited the most decline in HDP cases in the 20–24 age group. Specifically, the prevalence cases dropped from 42% to 13%, incidence cases decreased from 43% to 13%, mortality cases fell from 28% to 16%, and DALY cases reduced from 32% to 16%. In contrast, the High-income Asia Pacific region is the most significant increase in the 35–39 age group. The prevalence cases rose from 11% to 34%, incidence cases increased from 11% to 32%, mortality cases escalated from 14% to 25%, and DALY cases augmented from 12% to 30%. These observations suggest a shift in the distribution of HDP cases within WCBA, transitioning from younger to older age groups (Figure 4D and Supplementary Figures S6A–C).

Based on the aforementioned findings, it is evident that the prevalence cases, incidence cases, mortality cases, and DALYs cases of HDP exhibit distinct age-related patterns across different SDI regions. In low SDI regions, a higher burden of HDP is observed among younger age groups, with a notable decline as age increases. This trend may be attributed to the limited availability of medical resources and inadequate health education in these areas, which disproportionately affect younger women. Conversely, in high SDI regions, the burden of HDP is more pronounced among older age groups. This phenomenon may be explained by the presence of more complex underlying health conditions and the trend of delayed childbearing among older women in these regions.

### HDP burden associated with SDI

**Figure 5:**
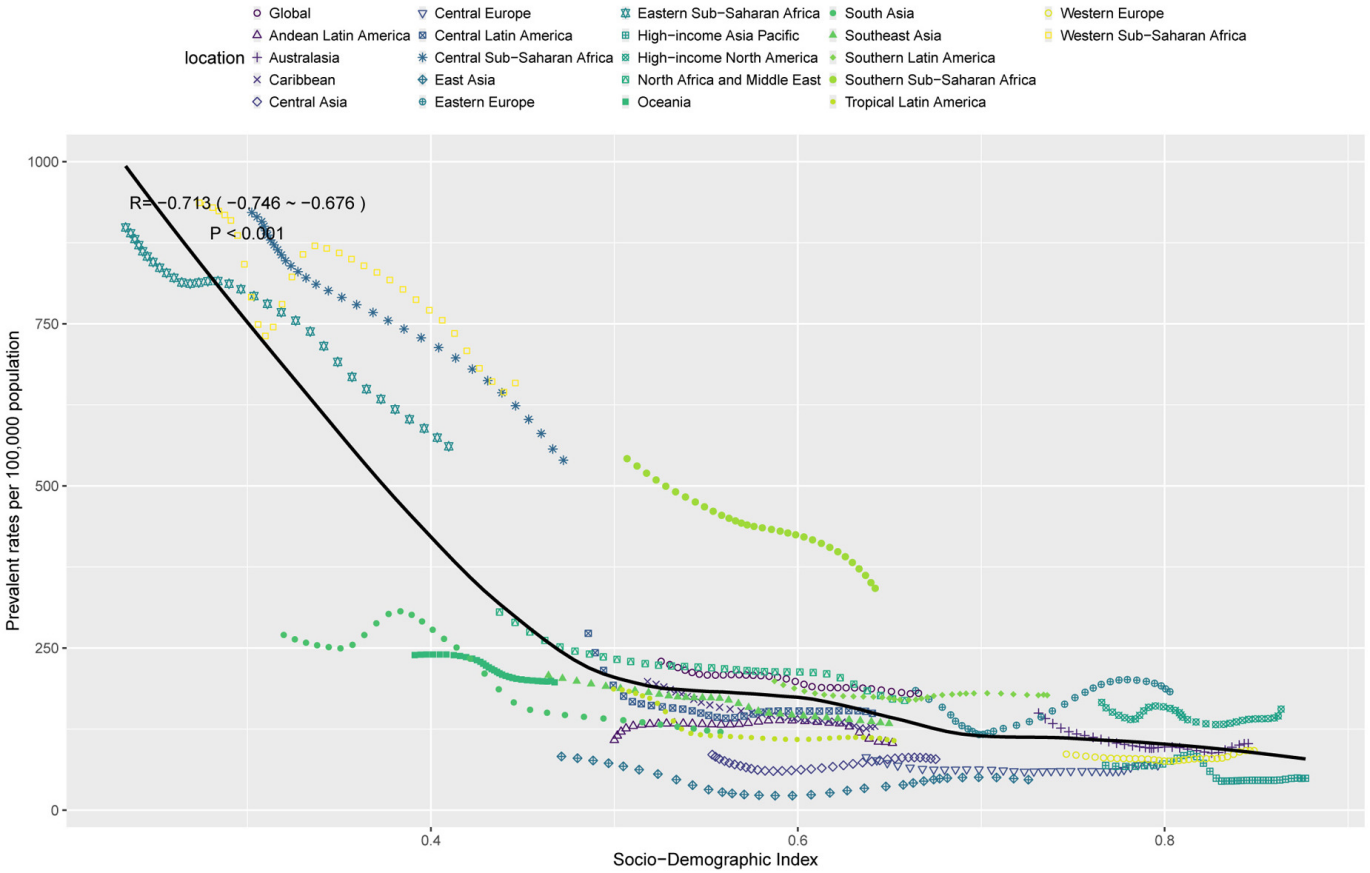
Correlation between SDI and prevalence rates per 100,000 population of HDP in WCBA across 21 GBD regions.

In 2021, a significant inverse correlation was observed between the SDI and the health metrics of HDP within the WCBA, including prevalence rates, incidence rates, mortality rates, and DALY rates. Across the 21 regions, the disease burden of HDP in WCBA remained relatively stable when the SDI value was between 0.5 and 0.7. Interestingly, the burden of HDP peaked at an SDI value of 0.8. Notably, regions such as Central Sub-Saharan Africa and Southern Sub-Saharan Africa exhibited a higher burden of HDP than anticipated. In contrast, areas like East Asia, Central Europe, and Central Asia had a lower burden than expected (Figure 5 and Supplementary Figures S7A–C). It indicated that the relationship between HDP burden and SDI is not a simple linear one. This complex relationship may be influenced by a combination of factors specific to each region, such as population structure, distribution of healthcare resources, lifestyle, and socio-economic conditions.

## Discussion

The GBD database analysis indicates a persistent global escalation in the incidence of HDP from 1990 to 2019, characterised by significant regional and age-specific disparities (3). However, the advent of the Coronavirus Disease 2019 (COVID-19) pandemic from 2019 to 2021 has posed tremendous challenges to delivering quality health services, with the potential impact on health outcomes still under investigation. For instance, COVID-19 infection during pregnancy can exacerbate the risk of severe maternal morbidity (19), subsequently leading to an elevated incidence of preterm births (20) and the necessity for cesarean deliveries (21). Additionally, the isolation imposed by the pandemic has been associated with a higher prevalence of postnatal depression among affected women (22–24). There is also evidence suggesting a potential link between maternal infections and the development of neurodevelopmental disorders in offspring (25). The infection results in prolonged hospital stays for mothers and infants (26) and escalates healthcare expenditure due to increased hospitalisation costs (27). Ongoing longitudinal studies further investigate these associations to elucidate the long-term implications of maternal COVID-19 infection on maternal and child health (28). Despite these adversities, the prevalence and morbidity of HDP in 2021 remained persistently elevated compared to 1990 levels, yet there was a notable reduction in patient fatalities and DALYs. This observed decline may be intricately linked to the targeted policy interventions implemented by the WHO, which have been instrumental even amidst the unprecedented health crisis of COVID-19.

Fan et al. observed a notable DALYs cases escalation among patients with D2M in regions characterised by middle-high and high SDI, signifying an augmented health burden in these locales (29). Conversely, the prevalence cases of childhood anaemia under five years of age exerts a considerable health burden, particularly in regions with lower SDI, where the economic implications of this condition are exacerbated (30). Asian women exhibit a heightened propensity for cancer, which subsequently amplifies the global health burden associated with this disease in lower SDI (31). Similarly, the level of SDI is negatively correlated with the prevalence cases, incidence cases, mortality cases and DALYs cases of HDP. The higher the SDI, the higher the economic and health care level, the better the care for HDP, and consequently, the lower the corresponding prevalence cases, incidence cases, mortality cases, and DALYs cases.

Over the past 32 years, the most significant increases in prevalence rates and DALY rates of HDP were showed in Western Sub-Saharan Africa and Andean Latin America, which may be attributed to social factors such as dietary habits (32, 33), industrialisation or urbanisation (34), inadequate health care services (35), and poor air quality (36). Our study also shows that prevalence and incidence are still increasing in most countries, but mortality and DALYs are decreasing in most countries. This suggests that, at the global level, healthcare and public health management efforts are positively affecting and reducing the causes of HDP.

Regarding age patterns, the global burden of HDP will be particularly severe in the 25–29 age group in 2021. In this age group, women in this stage of life have the highest fertility rates and the most significant burden. This highlights the importance of special attention and health management for this age group. The prevalence cases, incidence cases, mortality cases and DALYs cases of HDP have varied globally with age and economy over the last 32 years. The age group 30–39 years showed the most significant percentage change among them. In this case, the number of HDP cases transitioned from low to high age groups, showing a trend of increasing and decreasing first, and the peak of the number of cases showed a gradual backward shift in the age group with rising SDI. The higher the age of women in the reproductive years, the higher the complications of childbirth and the higher the prevalence of HDP. Advanced age (37) and economic disparities (38) are all significant risk factors for increased prevalence and morbidity. In economically developed regions, the implementation of standardized control strategies coupled with adequate financial investment enables effective intervention at the early stages of disease. This approach can effectively curb the further progression of illness, thereby preventing the exacerbation of the condition. Such early interventions not only contribute to the improvement of patients’ health outcomes but also significantly reduce the economic burden imposed by the disease on the region.

Currently, the global prevalence cases and incidence cases of HDP are still on the rise, while mortality cases and DALYs cases are on the decline. This indicates the progress of global medical technology and the effective implementation of public health interventions (39). In the future, it is necessary to continue strengthening global public health interventions. The global burden is uneven, with the level of SDI being the main reason (16). In the future, more attention needs to be paid to low SDI regions to improve the accessibility of medical resources. In low SDI regions, the burden is heavier among young women, while in high SDI regions, the burden is heavier among older women. Based on the above phenomenon, personalized policy-making and interventions are needed. For young women in low SDI regions, local policies should strengthen basic health care, increase the coverage of health education, and provide targeted maternal health services (40). For older women in high SDI regions, specialized medical services for older pregnant women should be provided, chronic disease management should be strengthened, and pre-pregnancy, pregnancy, and postpartum health consultations should be offered (41–43).

## Limitation

This study has several limitations. First, the estimates presented are not exhaustive, as HDP encompass various clinical subtypes with distinct symptoms, leading to potential misdiagnosis, particularly in economically underdeveloped regions where disease recognition is inadequate, resulting in an underestimation of the burden. Second, the GBD data, which this study relies on, heavily depends on modelling due to the absence of primary data in many countries. Furthermore, the global impact of COVID-19, including delayed births and constrained healthcare resources, may have compromised the accuracy of raw data.

## Conclusions

Overall, the global burden of HDP has exhibited a significant downward trend over the past 32 years. From the perspective of socio-economic factors, a negative correlation exists between DALYs burden and the SDI, regardless of specific regions or countries. The increase in disease burden is particularly pronounced in low SDI regions. In terms of age distribution patterns, the global burden of HDP peaked in the 25–29 age group in 2021. However, the most significant increase in HDP burden occurred in the 30–39 age group. Moreover, as SDI increases, the age group with the most substantial burden increase tends to shift towards older ages. Given these findings, it is imperative to implement targeted interventions based on the characteristics of different regions, economic statuses, and age groups in order to effectively mitigate the associated economic burden.

## Data Availability

The original contributions presented in the study are included in the article/Supplementary Material, further inquiries can be directed to the corresponding author.
