# Supplementary material for: Global, regional, and national trends and burden of hypertensive disorders in pregnancy among women of childbearing age from 1990 to 2021
Source: Front Glob Womens Health. 2025 May 9;6:1533843. doi: 10.3389/fgwh.2025.1533843 (PMC12098623; doi:10.3389/fgwh.2025.1533843)
Supplement: Supplementary file 1 [file Datasheet1.pdf]

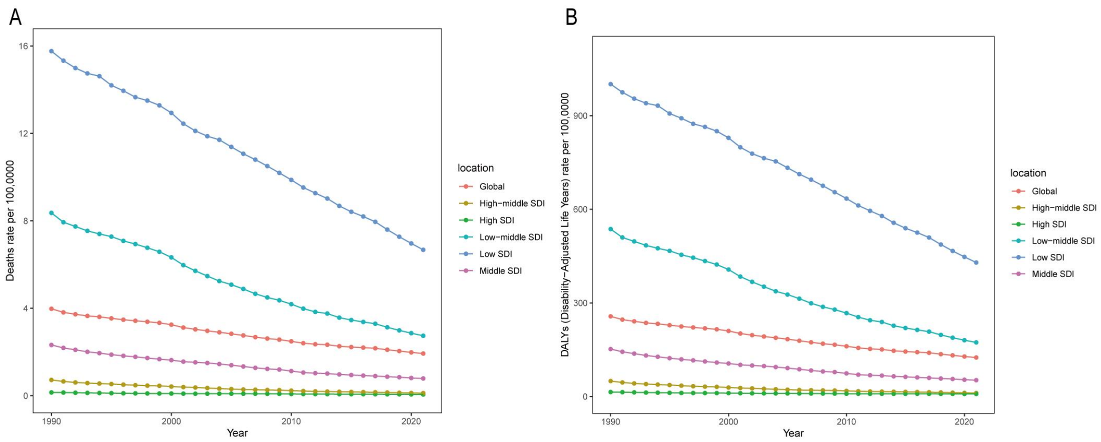

Supplement Figure 1. Global and regional temporal trends in HDP Burden in WCBA. (A) Mortality rates per 100,000 individuals from 1990 to 2021 in global and five selected territories. (B) DALY rates per 100,000 individuals from 1990 to 2021 in global and five selected territories.

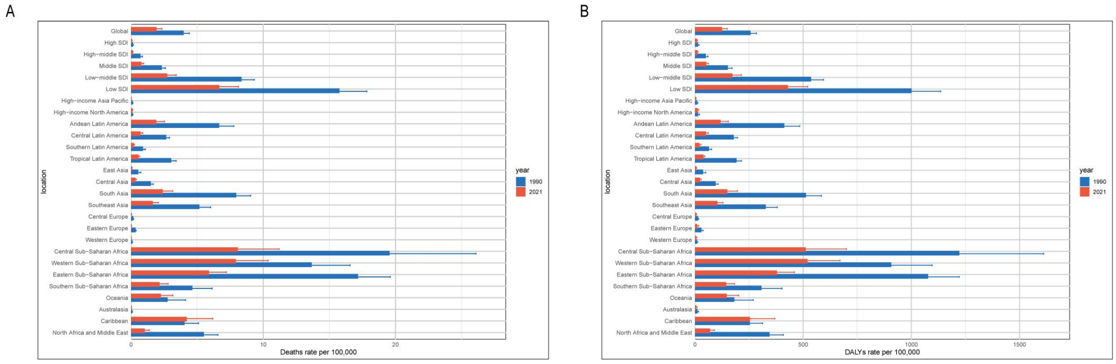

Supplement Figure 2. Regional temporal trends in HDP burden in WCBA. (A) Deaths rates per 100,000 population in 1990 and 2021. (B) DALYs rates per 100,000 population in 1990 and 2021.

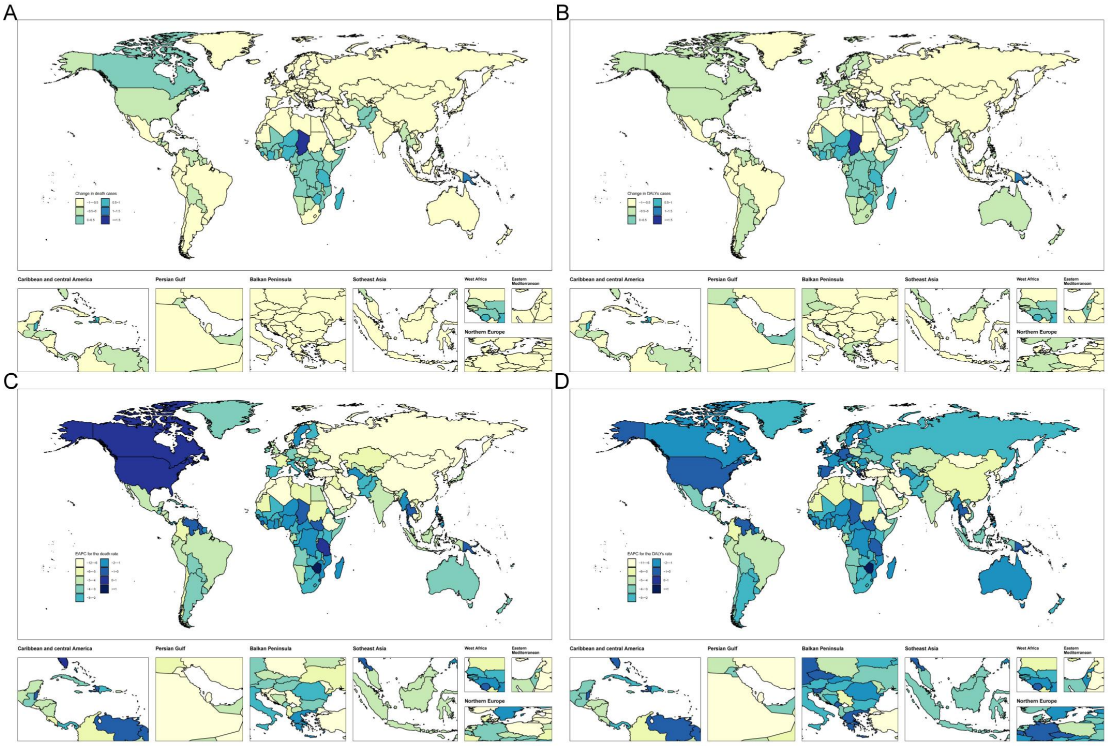

Supplement Figure 3. Global temporal trends in HDP burden in WCBA. (A) Percentage changes in death cases across 204 countries from 1990 to 2021. (B) Percentage changes in DALYs cases across 204 countries from 1990 to 2021. (C) EAPC in death across 204 countries from 1990 to 2021. (D) EAPC in DALYs across 204 countries from 1990 to 2021.

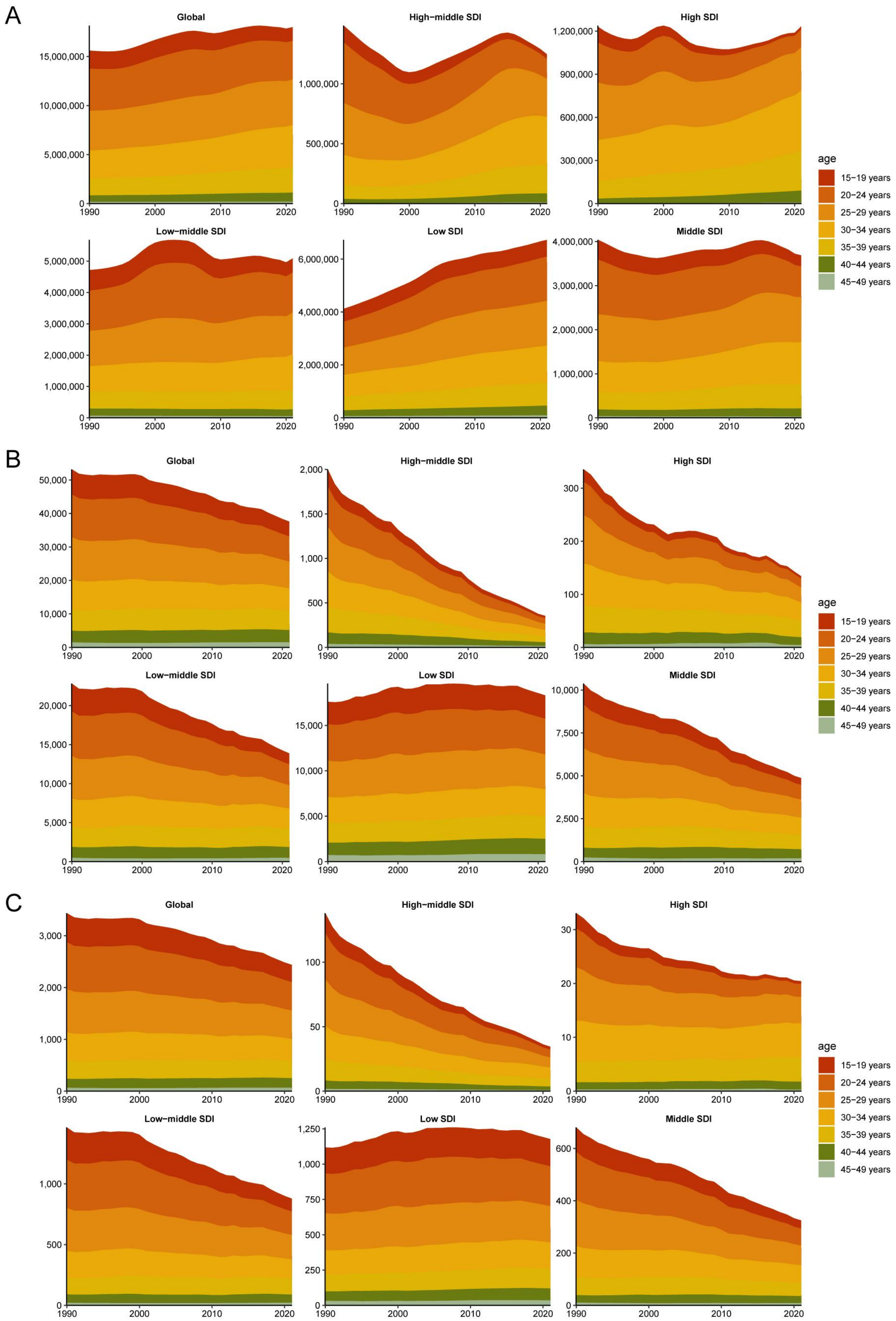

Supplement Figure 4. Trends in incidence, mortality, and DALYs of HDP across age groups (15-49 years) from 1990 to 2021. (A) Incidence cases across seven age groups (15-49 years) from 1990 to 2021, globally and in five territories with varying SDI. (B) Mortality cases across seven age groups (15-49 years) from 1990 to 2021, globally and in five territories with varying SDI. (C)

DALYs cases across seven age groups (15-49 years) from 1990 to 2021, globally and in five territories with varying SDI.

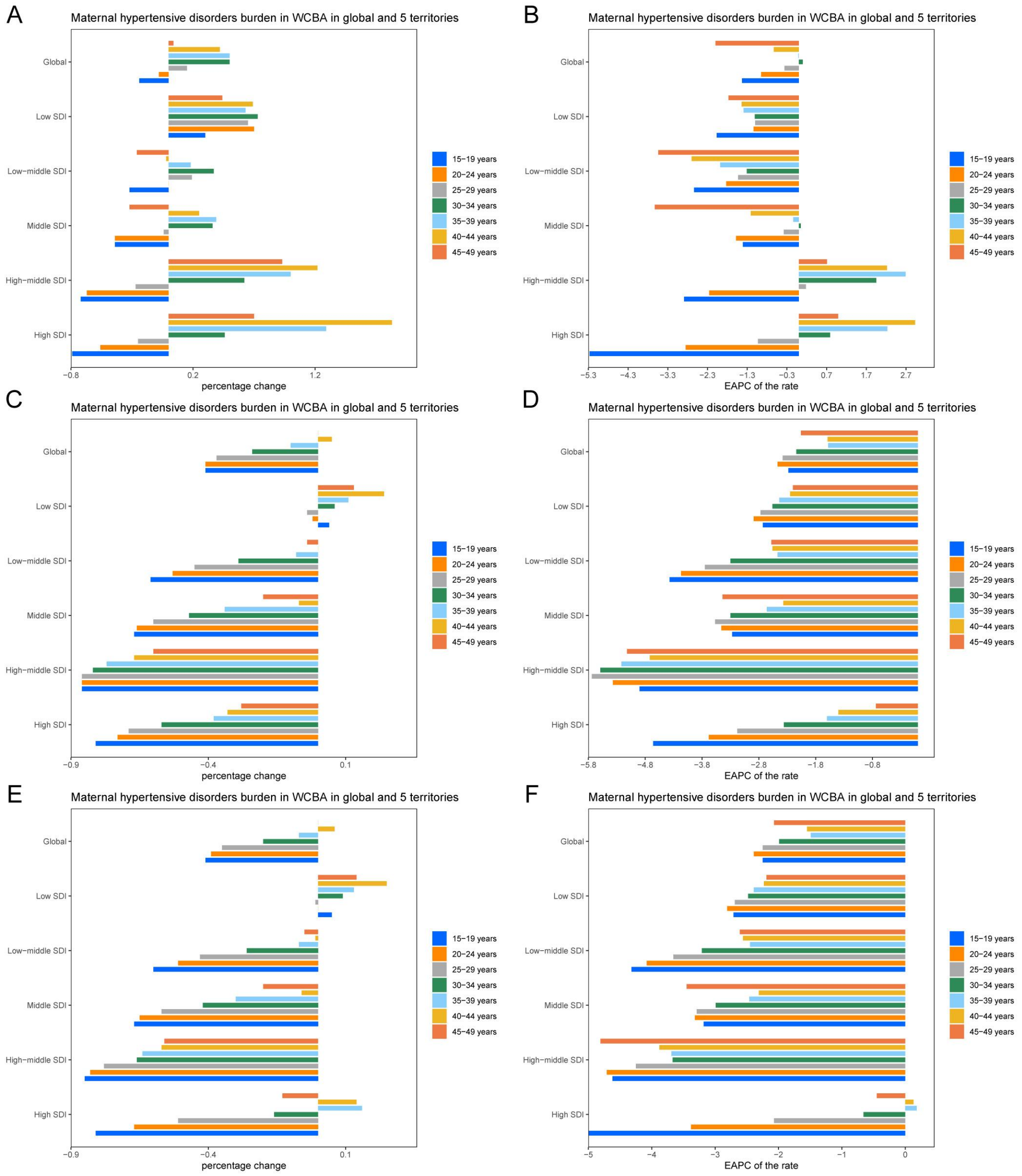

Supplement Figure 5. Age-patterned temporal trends in HDP burden in WCBA across different regions. (A) Percentage changes in incident cases across seven age groups, globally and in five territories, from 1990 to 2021. (B) EAPC in incident cases across seven age groups, globally and in five territories, from 1990 to 2021. (C) Percentage changes in mortality cases across seven age groups, globally and in five territories, from 1990 to 2021. (D) EAPC in mortality cases across seven age groups, globally and in five territories, from 1990 to 2021. (E) Percentage changes in DALYs across seven age groups, globally and in five territories, from 1990 to 2021. (F) EAPC in DALYs across seven age groups, globally and in five territories, from 1990 to 2021.

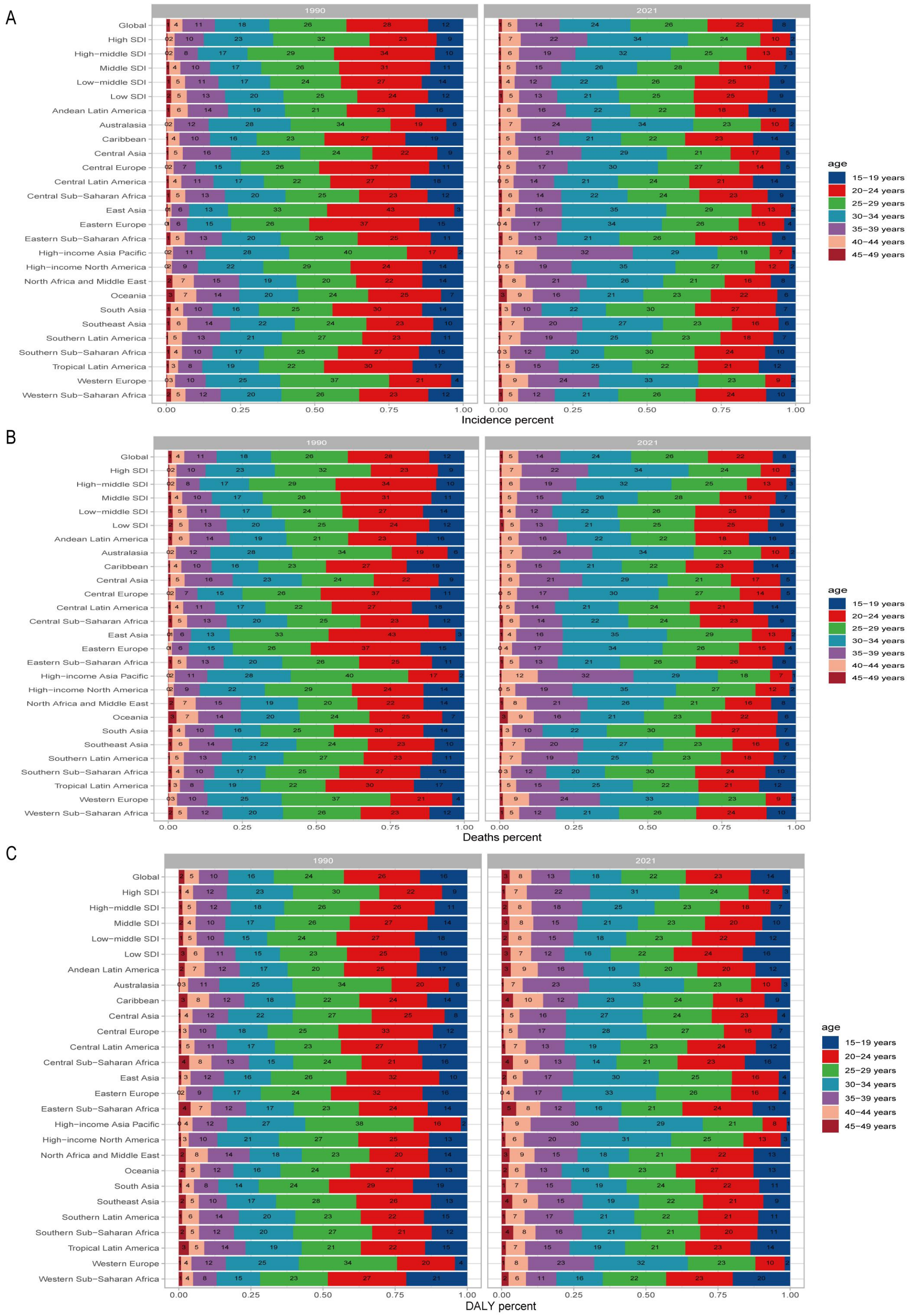

Supplement Figure 6. Distribution of HDP burden by age pattern in WCBA across different regions. (A) Distribution of incident cases across seven age groups as percentages, globally, in five territories, and 21 GBD regions, in 1990 and 2021. (B) Distribution of mortality cases across seven age groups as percentages, globally, in five territories, and 21 GBD regions, in 1990 and 2021. (C) Distribution of DALYs across seven age groups as percentages, globally, in five territories, and 21 GBD regions, in 1990 and 2021.

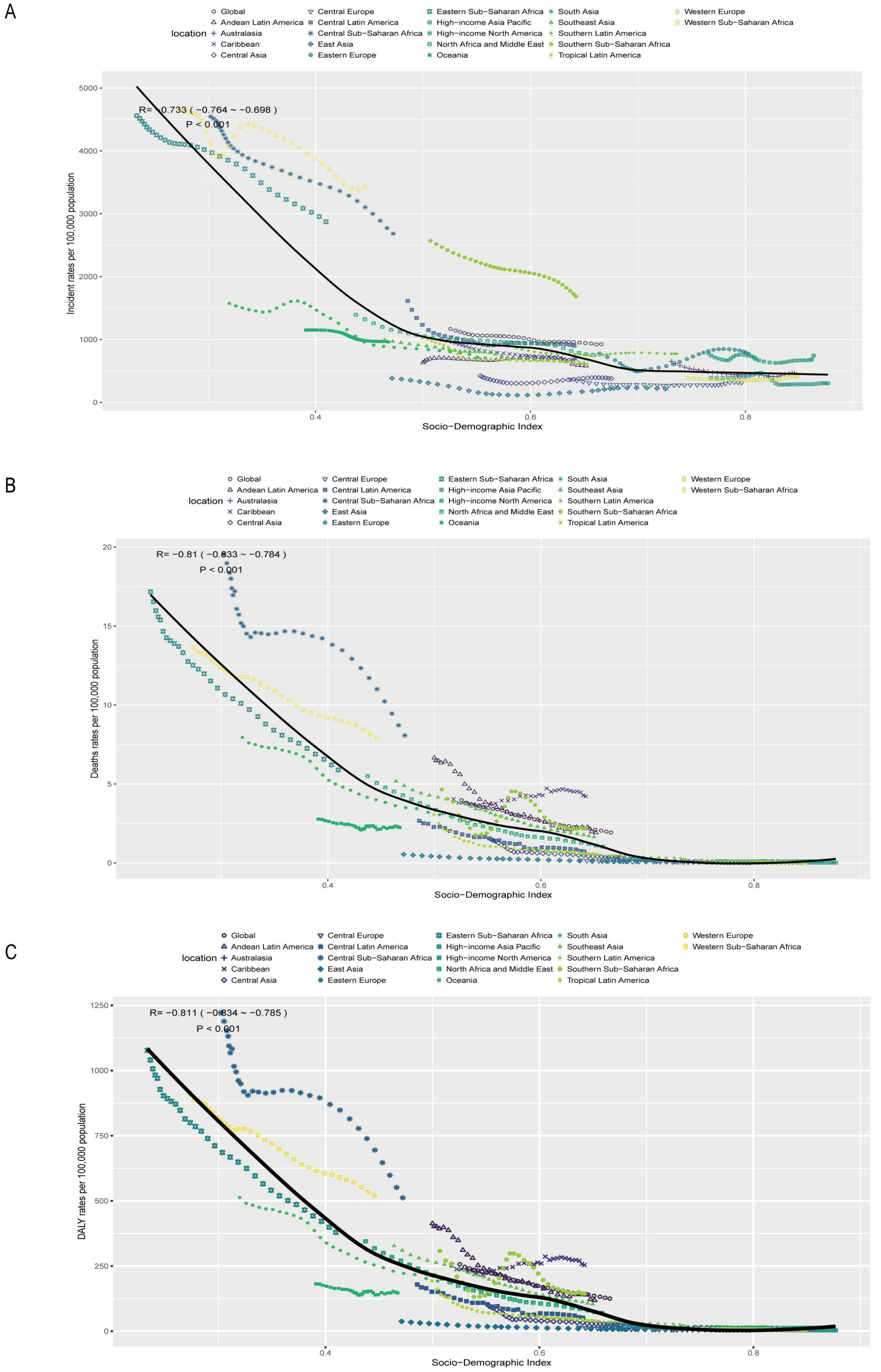

Supplement Figure 7. Correlation between SDI and incident (A), mortality (B), DALYs (C) rates

per 100,000 population of HDP in WCBA across 21 GBD regions.
